# Supplementary material for: SARS-CoV-2 PCR and antibody testing for an entire rural community: methods and feasibility of high-throughput testing procedures
Source: medRxiv. 2020 May 30:2020.05.29.20116426. Preprint. [Version 1] doi: 10.1101/2020.05.29.20116426 (PMC7273250; doi:10.1101/2020.05.29.20116426)
Supplement: Supplement 2020 [file 83633-2020.05.29.20116426-2.docx]

**Table 2.** **Supplies and quantities purchased to test approximately 1,900 participants**

| *Supplies* | *Quantities (n)** |
| --- | --- |
| **PPE supplies** | |
| Eye protection/face shields | 304 |
| Gloves | 900 (small)  1950 (medium)  6100 (large)  800 (extra-large) |
| Gowns/Tyvek suits | 154 |
| Respirators | 340 |
| Surgical masks (all volunteers, participants) | 2,850 |
| **Testing supplies** | |
| Alcohol wipes | 4,000 |
| Biohazard bags | 2,000 |
| Boxes/racks for microtainer storage | 10 |
| Gauze | 4,200 |
| Labels | 32,000 |
| Lancets | 4,000 |
| Microtainers | 2,000 |
| Synthetic swabs | 2,000 |
| Viral transport media | 2,000 |
| Tissues | 40 boxes |
| Bandaids | 4,000 |
| **Supplies needed to support operations** | |
| Biohazard waste/sharps containers | 8 |
| Chairs | 18 |
| Sanitizing wipes | 12 containers** |
| Cones (for spacing/distancing guidance) | Multiple |
| Cooler for transport | 2 |
| Garbage/garbage bags/waste management | 2 per lane |
| Generator(s) | 1 |
| Hand sanitizer | 40 bottles |
| Heat source (in-tent) | 2 |
| Mirror (for self-observed doffing PPE) | 2 |
| Plastic baskets (containing test kits) | 12 per lane |
| Refrigerators | 2 |
| Restrooms | 2 |
| Signage  Lane markers  “Slow 5 MPH”  “Warm your hands”  “Thank you for supporting your community!” | 30 |
| Tables | 4 per tent |
| Tents | 2 |
| Bins to organize supplies in tent | Multiple |
| Wi-Fi enabled tablets (check-in, registration) | 4 |
| Laptop computers and chargers | 4 |
| Wi-Fi hotspots | 3  (1 per tent, 1 at greeting station) |
| 2-way radios (onsite operations, leadership) | 4-6 |
| *Please note that not all supplies were completely consumed, and extra supplies donated to medical center.  **This is an estimate of need. Our operation was unable to procure sufficient quantity, so paper towels with spray disinfectant used when needed. | |
