# Supplementary material for: SARS-CoV-2 PCR and antibody testing for an entire rural community: methods and feasibility of high-throughput testing procedures
Source: medRxiv. 2020 May 30:2020.05.29.20116426. Preprint. [Version 1] doi: 10.1101/2020.05.29.20116426 (PMC7273250; doi:10.1101/2020.05.29.20116426)
Supplement: Supplement 2020 [file 83633-2020.05.29.20116426-3.docx]

**APPENDIX 1: Daily screening of study staff by email (based on UCSF Health’s daily screen)**

|  | **No** | **Yes** |
| --- | --- | --- |
| Have you been diagnosed with COVID-19 in the past 14 days? | If no, you may proceed to volunteer. | If yes, please stay home and notify Bolinas study leadership. |
| Do you live with someone who has been diagnosed with COVID-19 in the past 14 days? | If no, you may proceed to volunteer. | If yes, please stay home and notify Bolinas study leadership. |
| Have you had any of the following symptoms in the last 14 days (including the last 24 hours)?  - Fever over 100F - Sore throat - Difficulty breathing - Unexplained muscle aches - Cough - Loss of sense of smell or taste - Nasal congestion different from pre-existing allergies | If none of the above, you may proceed to volunteer. | If yes to *only* nasal congestion, it is possible you may proceed to volunteer. Please contact study leadership.  If yes to any other symptoms, please stay home and notify Bolinas study leadership. |
| Have you been in unprotected contact with someone diagnosed with COVID-19 in the past 14 days?  (unprotected means without full PPE at work or *close* contact in the community) | If no, you may proceed to volunteer. | If yes, please stay home and notify Bolinas study leadership. |
| Have you returned from travel outside the US or from NY/NJ/CT in the past 14 days? | If no, you may proceed to volunteer. | If yes *and* symptoms, please stay at home and notify Bolinas study leadership.  If yes and NO symptoms, you may proceed to volunteer with self-monitoring of symptoms twice daily. |
